# Analysis of gut microbiota diversity in Hashimoto’s thyroiditis patients

**DOI:** 10.1186/s12866-022-02739-z

**Published:** 2022-12-24

**Authors:** Jilai Liu, Xuejun Qin, Boxi Lin, Jing Cui, Juan Liao, Fu Zhang, Qing Lin

**Affiliations:** Department of Clinical Laboratory, People’s Hospital Affiliated of Fujian University of Traditional Chinese Medicine, Fuzhou, 350004 China

**Keywords:** Hashimoto’s thyroiditis, Gut microbiota, 16S rDNA, High-throughput sequencing, Biomarkers

## Abstract

**Background:**

Hashimoto’s thyroiditis (HT) is an autoimmune disease. Recent studies have found that the gut microbiota may play an important role in inducing HT, but there are no systematic studies on the changes in the gut microbiota during the development of HT.

**Methods:**

In this study, 16S rDNA high-throughput sequencing technology in combination with the Kruskal–Wallis test, CCA/RDA analysis, Spearman correlation analysis, and other statistical methods were used to analyze the effects of age, gender, hormones, and other environmental factors on gut microbiota by comparing the differences in the microbiota at different stages of HT development.

**Results:**

The results showed that there were differences in the gut microbiota composition between healthy people (HCA) and in patients with HT. *Lachnoclostridium*, *Bilophila,* and *Klebsiella* were enriched in the HCA group, while *Akkermansia*, *Lachnospiraceae*, *Bifidobacterium*, *Shuttleia,* and *Clostriworthdia* were enriched in the HT group. Environmental factors analysis revealed that the *Bifidobacterium* and *Klebsiella* were two groups of bacteria that have undergone dramatic changes in HCA and HT, and mainly affected by gender. *Romboutsia* and *Haemophilus* regulated by the hormone of free triiodothyronine (FT3) may promote the development of HT, while *Faecalibacterium* and *Lachnospiraceae* regulated by free thyroxine (FT4) may protect the host.

**Conclusions:**

Comprehensive studies have shown that gender is an important factor affecting gut microbial composition, but with the development of HT, hormones, age, and TSH begin to become dominant factors.

**Supplementary Information:**

The online version contains supplementary material available at 10.1186/s12866-022-02739-z.

## Introduction

Hashimoto’s thyroiditis (HT) is an organ-specific autoimmune disease with an incidence of 0.3‰ to 1.5‰, and the incidence in female is 4 to 10 times that of male [1]. The disease, which was first reported by Hakaru Hashimoto in 1912 [2], is characterized by chronic inflammation and is accompanied by tertiary lymphoid follicle development and increased circulating autoantibody concentrations against thyroid peroxidase (anti-TPO) and thyroglobulin (anti-TG) [3]. HT is usually asymptomatic, and the most common presentation is goiter, which may present with or without hypothyroidism. During HT, patients may experience subclinical hypothyroidism or even significant hypothyroidism [4]. Although the etiology and mechanism of HT are unclear, it is generally accepted that it is caused by the interaction between genetic susceptibility factors, epigenetic effects, and various environmental factors [5]. Several twinstudies have provided epidemiological evidence for genetic susceptibility to HT, and the concordance rate of monozygotic twins with HT is higher than that of dizygotic twins [6, 7]. In addition to genetic factors, environmental factors such as excessive iodine intake [8], vitamin D deficiency [9], selenium deficiency, Helicobacter pylori infection, viral infection (type C hepatitis virus, human parvovirus B19, and enterovirus), hygiene, and new treatment modalities and chemical agents also play an important role in the occurrence and development of HT [10].Recently, the relationship between gut microorganisms and HT has attracted the attention of researchers.

A large number of commensal microorganisms inhabit the human gut, with the total amount of microorganisms being dozens of times higher than the number of human cells, with the number of encoded genes being 100 times that of the human genome [11]. As such, the gut microbiota is considered to be the second set of genetic codes outside of a human’s own genome [12]. Humans and complex, dynamic microbial communities co-evolve in symbiotic relationships [13]. The microbiota plays a key role in maintaining gut homeostasis: a lack of microbiota leads to changes in the gut barrier, such as a reduced overall gut surface area, shortened villi and gut crypts, increased gut permeability, and qualitative and quantitative changes in the mucus layer [14]. In recent years, numerous studies have elucidated the critical role of the gut microbiota in various metabolic and autoimmune diseases, such as obesity [15], diabetes [16, 17], systemic lupus erythematosus (SLE) [18], rheumatoid arthritis [19], etc. Köhling HL et al. proposed that the gut microbiota may play an important role in the induction of HT [20].There is much evidence that gut dysbacteriosis, bacterial overgrowth, and increased gut permeability favor the development of HT, and it has been suggested that the thyroid–gut axis may influence our overall metabolism [21, 22]. First, dysbacteriosis leads to impaired gut microbiota and increased intestinal permeability, allowing antigens to enter circulation and activate the immune system [23]. Second, the metabolites of the microbiota can activate pro-or anti-inflammatory programs [24]. Third, microorganisms influence thyroid hormone levels by regulating iodine uptake, degradation, and enterohepatic circulation [21].. In addition, the microbiota may play a role in thyroid disease by affecting the production of neurotransmitters, the hypothalamic–pituitary axis, and dopamine, which, in turn, affects the secretion of TSH [21]. Gut microbiota metabolites, particularly short-chain fatty acids (SCFAs), can act as energy sources for enterocytes and, together with thyroid hormones, enhance intestinal epithelial cell differentiation and enhance tight intercellular connections [25]. The gut microbiota produces secondary bile acids in the colon, which can interfere with TSH levels to regulate energy metabolism.

Although there have been many studies on the effects of the gut microbiota on host thyroid hormone synthesis and secretion as well as on the potential association between gut microbiota and HT, there have been no systematic studies on the gut microbiota trends during the gradual transition from healthy people to different stages of HT development or on the correlation between changes in the gut microbiota during this process and different environmental factors, such as age, gender, and hormones. In this study, 16S rDNA high-throughput sequencing was carried out on gut microbes from a large number of samples and was combined with statistical analysis to try to clarify the following two questions: (1) Do gut microbes differ between healthy people and HT patients, and does this difference increase with the development of HT? (2) Are gut microbes associated with different environmental factors, such as age, gender, hormones, etc.? What is the corresponding relationship between the microbiota and environmental factors? Clarifying these scientific issues will help us to better understand the occurrence and development of HT, and will provide a scientific basis for better promoting the intervention and clinical treatment of HT.

## Materials and methods

### Study population and specimen collection

From February 2021 to March 2022, 89 participants recruited from the Department of Endocrinology and the Department of Preventive Medicine of the People’s Hospital Affiliated of Fujian University of Traditional Chinese Medicine participated in our study. The study population consisted of 42 healthy volunteers (21 males and 21 females) as controls; 37 HT patients with normal thyroid function (11 males and 26 females); and 10 HT patients with thyroid dysfunction (5 males and 5 females).HT diagnosis was defined as follows: (1) elevated serum TPOAb and/or TGAb and (2) morphologic features of HT in the thyroid gland on ultrasound. Exclusion criteria consisted of known thyroid diseases (including inflammation, nodules, dysfunction, etc.), tumors, blood diseases, inflammatory diseases (infectious and non-infectious), liver and kidney insufficiency, hypertension, diabetes, dyslipidemia, and the use of lipid regulation medication. Those who had taken thyroid-impairing medication within the past 3 months, and those who had taken antibiotics in the week before stool samples were also excluded. According to the attributes of the sample and the development stage of the disease, we divide the sample into the following groups: HCA: healthy population, HT1A: Hashimoto’s thyroiditis with normal thyroid function, HT2A: Hashimoto’s thyroiditis with abnormal thyroid function, HCM: healthy male population, HCF: healthy female population, HT1M: Hashimoto’s thyroiditis in normal thyroid males, HT1F: Hashimoto’s thyroiditis in normal thyroid females, HT2M: Hashimoto’s thyroiditis in abnormal thyroid males, HT2F: Hashimoto’s thyroiditis in abnormal thyroid females. The venous blood samples were collected from all of the subjects in the morning following an overnight fast. Fecal samples were collected in tubes pre-loaded with fecal DNA stabilizer, were transported to our laboratory, and were stored at -80 °C until further processing.

### Thyroid function and antibody testing

Thyroid is a very important endocrine organ of vertebrates. It regulates metabolism, growth rate and other system functions of the body by producing thyroxine, free triiodothyronine (FT3) and free thyroxine (FT4). We use the Thyroid-stimulating hormone (TSH), FT3, FT4, thyroid peroxidase antibody (TPOAb), and thyroglobulin antibody (TGAb) to evaluate the thyroid function. The increase of TPOAb and TGAb is a sign of thyroid destruction. The increase of TPOAb and TGAb leads to the decrease of FT3 and FT4, and the increase of TSH, which leads to HT. The Peripheral blood (5 mL) was collected from all subjects in the morning after overnight fasting (≥ 8 h) and stored in procoagulant tubes. Serum was isolated at 3500 rpm for 10 min, then the TSH, FT3, FT4, TPOAb, and TGAb were measured via chemiluminescence (Abbott Diagnostics, Chicago, Illinoils, USA). Reference ranges were TSH: 0.35–4.94 mIU/L, FT3: 2.63–5.70 pmol/L, FT4: 9.01–19.05 pmol/L, TPOAb: 0–5.61 IU/mL, and TGAb: 0–4.11 IU/mL.

### Sample DNA extraction

Gut bacterial DNA was extracted using the E.Z.N.A.® Soil DNA Kit (Omega Bio-Tek, Norcross, GA, USA), and the extraction method used was as described in the kit instructions. DNA purity and concentration were checked by Nanodrop2000 (Thermo Fisher Scientific, Waltham, Massachusetts, USA), and DNA integrity was checked by agarose gel electrophoresis.

### PCR amplification

Primer corresponding region: 16S V3-V4 region. 338F: ACTCCTACGGGAGGCAGCAG, 806R: GGACTACHVGGGTWTCTAAT. (1) PCR amplification system:5 × FastPfu Buffer 4 µL, 2.5 mMdNTPs 2 µL, upstream primer (5 µM) 0.8 µL, downstream primer (5 µM) 0.8 µL, FastPfu DNA Polymerase 0.4 µL, BSA 0.2 µL, genomic DNA 10 ng, and supplemented with ddH2O to 20 µL. (2) PCR amplification conditions: pre-denaturation at 95 °C for 3 min, 30 cycles of denaturation for 30 s at 95 °C, annealing for 30 s at 55 °C, 45 s at 72 °C for elongation, and a final extension at 72 °C for 10 min.

### High-throughput sequencing

PCR products were quantified with a Quantus™ Fluorometer. According to the sequencing volume requirements of each sample, the corresponding proportions were mixed. Libraries were constructed using the NEXTFLEX Rapid DNA-Seq Kit (PerkinElmer, Waltham, Massachusetts, USA) and were sequenced using MiSeq PE300 in Illumina.

### Data optimization

For quality control of the raw sequencing sequences, fastp (https://github.com/OpenGene/fastp, version 0.20.0) software [26] was used. FLASH (http://www.cbcb.umd.edu/software/flash Version 1.2.7) software [27] was used for splicing: bases with a tail quality value of less than 20 reads were filtered to remove reads containing N bases; pairs of reads were spliced (merged overlap) into a sequence according to the overlap relationship between the PE reads with a minimum length of 10 bp. The maximum mismatch ratio allowed in the overlap region of the spliced sequence was 0.2 to screen for nonconforming sequences. Samples were distinguished according to the barcode and primers at the beginning and end of the sequence, and the sequence direction was adjusted, with the barcode allowing 0 mismatches and a maximum primer mismatch of 2.

### Operational taxonomic unit (OTU) cluster analysis

Using the UPARSE software (http://drive5.com/uparse/, version 7.1) [28], the sequences were clustered for OTUs when the similarity was 97%, and the specific process was as follows: non-repetitive sequences were extracted from the optimized sequences to remove single sequences without repeats; OTU clustering was performed for non-repetitive sequences (excluding single sequences) when there was 97% similarity; chimeras were removed during the clustering process to obtain OTU representative sequences; all optimized sequences were mapped to OTU representative sequences, and sequences with more than 97% sequence similarity to the OTU representative sequences were selected to generate OTU tables. In order to minimize the impact of the sequencing depth on subsequent diversity analysis, the number of sequences in all of the samples was flattened to 20,000, and after flattening, the average sequence coverage (Good’s coverage) of each sample was still able to reach 99.09%. The RDP classifier(http://rdp.cme.msu.edu/, version 2.11) [29] was used to align the Silva 16S rRNA gene database (v138) for the taxonomic annotation of OTU species, achieving a confidence threshold of 70%, and the community composition was counted for each sample at different species taxonomic levels. Next, 16S functional prediction analysis was performed using PICRUSt2 (version 2.2.0) software [30].

### Data analysis

All the data analyses were performed on the Major Bio Cloud platform (https://cloud.majorbio.com). The details are as follows: using the mothur software (http://www.mothur.org/wiki/Calculators) [31], Alpha diversity knowledge Chao 1, Shannon index, etc., were calculated, and the Wilcoxon rank sum test was used to analyze group differences in Alpha diversity. Partial least squares discriminant analysis (PLS-DA) was used to group samples, and linear discriminant effect size (LEfSe) analysis (http://huttenhower.sph.harvard.edu/LEfSe) [32] was used to identify bacterial groups that differ significantly in the genus-level abundance between groups, the kruskal–wallis test followed by Tukey–kramer post-hoc test was used for comparative analysis of multiple groups of samples, and then the Wilcoxon based method was used for pairwise comparative analysis. Environmental factor association analysis was performed using redundancy analysis (RDA)/canonical correspondence analysis (CCA) to analyze the effects of gender, age, and hormones on the community structure of gut microbes. Spearman correlation analysis, which visualizes the relationship between different species in a sample and environmental variables, assesses the correlation between microbial classification and environmental variables. Functional predictions of the gut microbiota were performed using Phylogenetic Investigation of Communities by Reconstruction of Unobserved States (PICRUSt) [33] to get theKyoto Encyclopedia of Genes and Genomes (KEGG) analysis results, especially in the analysis of metabolic function, the KEGG orthology (KO) were used for the analysis (www.kegg.jp/kegg/kegg1.html) [34–36].

## Results

### 16S rDNA Sequencing analysis of gut microbes in patients with Hashimoto’s thyroiditis

In this study, 16S rDNA sequencing analysis was performed on 89 samples. Through that analysis, 4,295,693 optimized sequences, 1,772,947,074 bases, an average sequence length of 412 bp, and a total of 912 OTUs were obtained. The following statistics were obtained for the species annotation results: Domain: 1, Kingdom: 1, Phylum: 12, Class: 17, Order: 49, Family: 92, Genus: 283, and Species: 602 (Tables S1 and S2). The TOP5 phylum-level species were as follows: *Firmicutes*, *Bacteroidota*, *Actinobacteriota*, *Proteobacteria*, and *Fusobacteriota*, and the TOP5 genus-level species were *Bacteroides*, *Blautia*, *Faecalibacterium*, *Bifidobacterium*, and *Escherichia-Shigella* (Table S2).

**Fig. 1.**
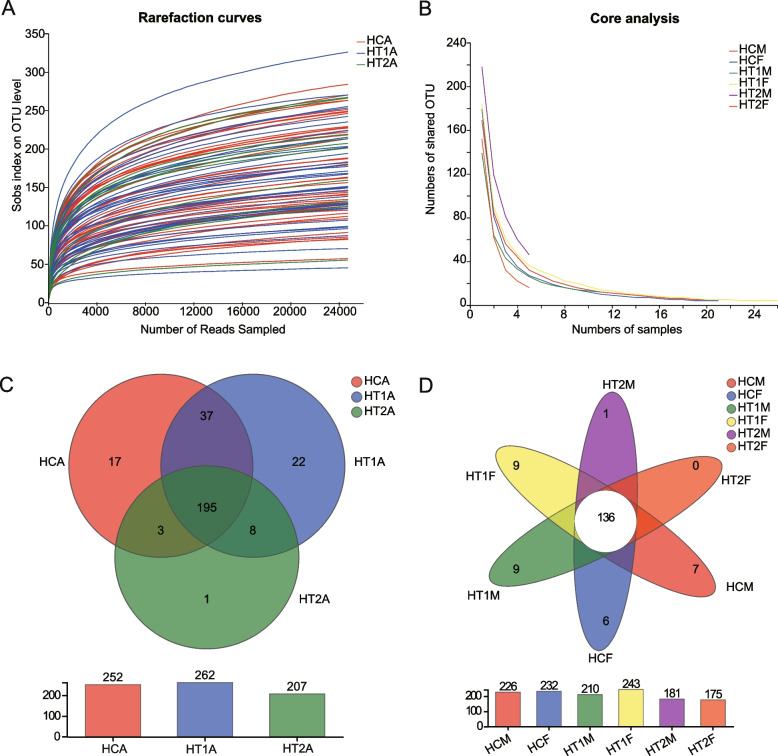
16S rDNA sequencing analysis of gut microbes in patients with Hashimoto’s thyroiditis. **A** Rarefaction curves analysis. **B** Pan/Core core species analysis. **C** Venn diagram analysis of gut microbes in healthy people and patients with Hashimoto’s thyroiditis. **D** Venn diagram analysis of gut microbes in healthy people and patients with Hashimoto’s thyroiditis. HCA: healthy population, HT1A: Hashimoto's thyroiditis, people with normal thyroid function, HT2A: Hashimoto’s thyroiditis, people with abnormal thyroid function, HCM: healthy male population, HCF: healthy female population, HT1M: Hashimoto’s thyroiditis in normal thyroidmales, HT1F: Hashimoto’s thyroiditis in normal thyroid females, HT2M: Hashimoto’s thyroiditis in abnormal thyroid males, HT2F: Hashimoto’s thyroiditis in abnormal thyroid females

Rarefaction curve analysis showed that the curves of each sample were flat, and the sequencing depth was sufficient to reflect the species diversity in the samples (Fig. 1A). The core species analysis showed that as the number of different samples increased, the number of shared core OTUs gradually decreased. In addition, the curves also showed that the number of sequencing samples in the HT2A group was less than that in the HCA and HT1A groups (Fig. 1B). Although Alpha diversity analysis showed no significant differences among HCA, HT1A, and HT2A (Table S3), a Venn diagram analysis based on HCA, HT1A, and HT2A showed that the number of species in the HT2A group was the lowest among the three groups of samples, with 195 OTUs being common to the three groups. According to the analysis, 17 OTUs were unique to HCA, 22 were unique to HT1A, and only 1 was unique to HT2A. It was also determined that HCA and HT1A had 37 OTUs in common, with 3 of those OTUs being shared withHT2A, and there were 8 OTUs in common with HT1A and HT2A, which showed that HCA was closer to HT1A, while the HT1A and HT2A species were more similar to each other (Fig. 1C).After further subdividing the sequencing samples into male and female, Wenn analysis found that there were 136 OTUs in the six groups, with 7 OTUs being unique to HCM, 6 being unique to HCF, 9 being unique to HT1M, 9 being unique to HT1F, 1 being unique to HT2M, and 0 being unique to HT2F, indicating that the gut microbiota differed between genders and that subsequent analyses needed to consider the effect of gender on the results (Fig. 1D).

### Analysis of gut microbial composition and differences in patients with Hashimoto’s thyroiditis at different developmental stages and genders

**Fig. 2:**
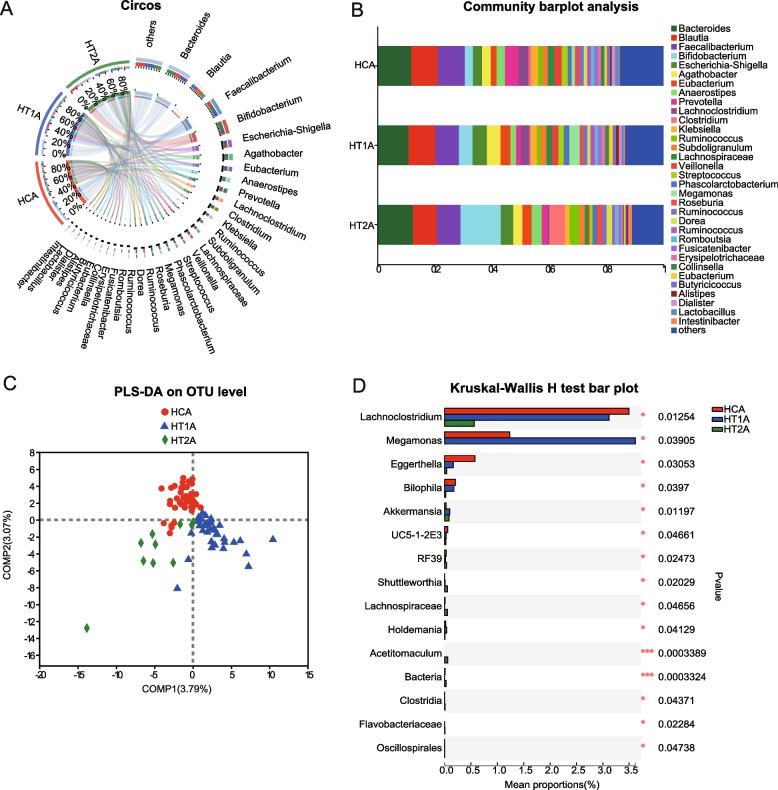
Based on gut microbial composition and differential analysis in patients with Hashimoto’s thyroiditis at different stages of development. **A** Circos sample and species relationship diagram: The left half circle represents the species composition in the sample; the different color of outer ribbon: different samples (HCA, HT1A, HT2A); inner ribbon: bacteria species; the length of the ribbon: the abundance of the corresponding species. The right half circle: the bacteria abundance in different samples; the outer ribbon: bacteria species; the inner ribbon: different samples (HCA, HT1A, HT2A), length of the ribbon: the abundance of the corresponding bacteria species. **B** Analysis of gut microbial community composition of different samples: The ordinate is the sample name; the abscissa is the proportion of a species in the sample; the different-colored columns represent different species; and the length of the column represents the proportion of a species. **C **PLS-DA sample group analysis: The dots with different colors or shapes represent the sample groups under different environments or conditions, and Comp1 and Comp2 represent the suspected influencing factors affecting the deviation in the microbial composition of the two groups of samples. **D** Analysis of the significant differences between groups based on the community abundance data in the sample and the Kruskal–Wallis rank sum test: The Y-axis represents the species names at certain taxonomic levels; the X-axis represents the average relative abundance of species in different groups; and different-colored bars indicate different grouping. The *p* values are on the far right, where *p ≤ 0.05 and ***p ≤ 0.001

**Fig. 3:**
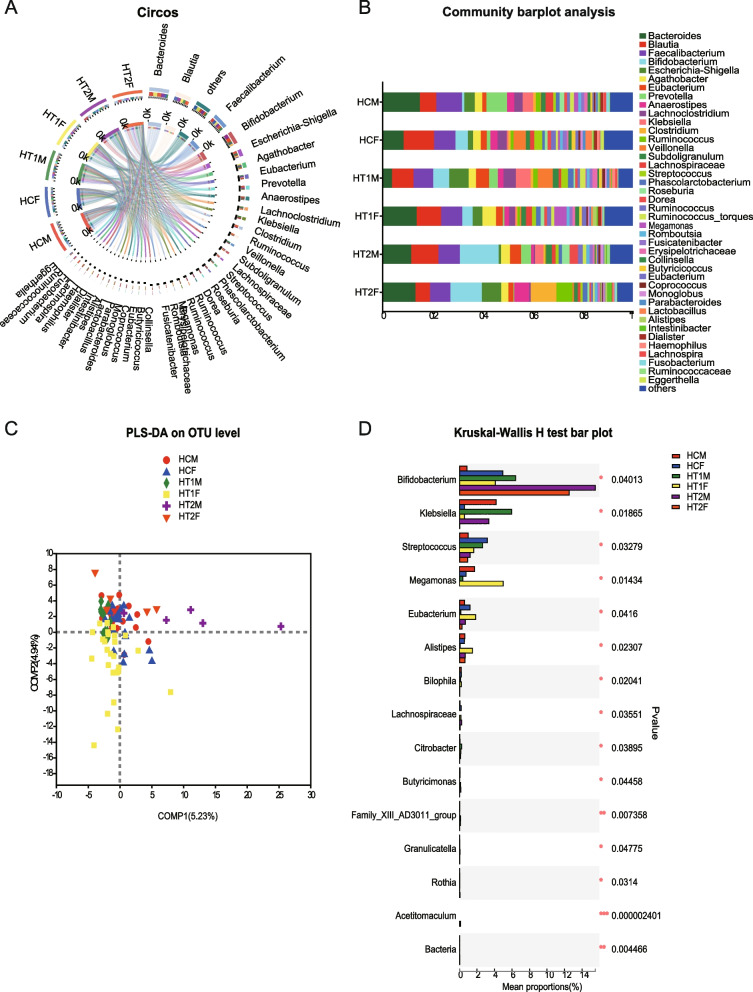
Gender-based analysis of gut microbial composition and differences in Hashimoto’s thyroiditis patients. **A** Circos sample and species relationship diagram: The left half circle represents the species composition in the sample; the different color of outer ribbon: different samples (HCM, HCF, HT1M, HT1F, HT2M, HT2F); inner ribbon: bacteria species; the length of the ribbon: the abundance of the corresponding species. The right half circle: the bacteria abundance in different samples; the outer ribbon: bacteria species; the inner ribbon: different samples (HCM, HCF, HT1M, HT1F, HT2M, HT2F), length of the ribbon: the abundance of the corresponding bacteria species. **B** Analysis of the community composition of different samples of gut microbes based on different genders: The ordinate is the sample name, the abscissa is the proportion of the species in the sample, the different-colored columns represent different species, and the length of the column represents the proportion of a species. **C** PLS-DA grouping analysis of gut microbial samples based on different genders: Points with different colors or shapes represent sample groups under different environments or conditions, and Comp1 and Comp2 represent the suspected influencing factors responsible for the deviation in the microbial composition of the two groups of samples. **D** Significant differences between groups based on the community abundance data in the samples and the Kruskal–Wallis rank sum test in different gender groups: The Y-axis represents the species names at a certain taxonomic level, and the X-axis represents the average relative value of the species in the different groups. Abundance is represented by different-colored bars, which indicate different groupings; *p* values are located on the far right. *p ≤ 0.05, **p ≤ 0.01, ***p ≤ 0.001

Microbial diversity analysis showed that HCA and HT1A had similar species compositions, while HT2A was different from the former two, and it was very interesting that *Bifidobacterium* also gradually increased in abundance as HT deepened (Fig. 2A and B).An analysis of the PLS-DA sample groupings showed that the three groupings were well distinguishable, indicating that the gut microbes differed in composition between healthy people and patients, and this difference was reinforced as the disease severity increased (Fig. 2C). Comparative analysis of multiple groups of samples based on Kruskal–Wallis test showed that multiple species were significantly different between the different groups, among which *Lachnoclostridium* and *Holdemania*were enriched in the HCA and HT1A groups (*p* ≤ 0.05); *Akkermansia* was enriched in HT1A and HT2A (*p* ≤ 0.05); *Eggerthella* and *Bilophila* were significantly enriched in the HCA groups (*p* ≤ 0.05); *Ralstonia*, *Fournierella*, and *Megamonas* were enriched in HT1A (*p* ≤ 0.05); and *Acetitomaculum*, *Shuttleworthia*, *Flavobacteriaceae*, unclassified_c__*Clostridia*, *Lachnospiraceae*_NC2004_group, and unclassified_o__*Oscillospirales* were enriched in HT2A (*p* ≤ 0.05) (Fig. 2D). In order to verify the results of comparative analysis of multiple groups of samples, the Wilcoxon based method was used for pairwise comparative analysis between samples, and further supported the above results Figure S1-S12. Furthermore, after analyzing the samples by distinguishing between males and females, it was found that there were differences in the species composition and abundance between genders, and this difference was strengthened as HT intensified (Fig. 3A and B). PLS-DA analysis showed that the males and females in the HCA group were relatively concentrated and that there was less discrimination, but the HT1A and HT2A groups showed a discrete nature, indicating that HT leads to the isolation of gut microbes in both males and females (Fig. 3C). The significance analysis of species differences revealed that *Bifidobacterium* was much more abundant in females than in males in the HCA group, but this was reversed in both HT1A and HT2A in the HTA group, with higher abundances in males than in females, and its overall abundance increased with the degree of HT (*p* ≤ 0.05). The levels of *Klebsiella* bacteria were higher in males than in females and showed an initial increasing trend and then a decreasing trend with the aggravation of the HT level, especially in the HT2A group, where HT2AF was significantly down-regulated (*p* ≤ 0.05). *Megamonas* was higher in males than in females in the HCA group, and this was reversed in the HT1A group, and its abundance was the highest in HT1F group (*p* ≤ 0.05). *Streptococcus* levels were much higher in females than in males in the HCA group, but this was reversed in HT1A and HT2A, and the levels were significantly decreased in HT2A (*p* ≤ 0.05) (Fig. 3D). This result indicated that HT not only caused differences in the gut microbiota between males and females, but that as the degree of disease severity intensified, the trend observed in the gut microbiota changes was further strengthened. Interestingly, gender plays an important role in the composition of gut microorganisms. LEFSe analysis further supported the above results (Figure S13-S17).

### Analysis of the effect of differences in gut microbes on function in patients with Hashimoto’s thyroiditis

**Fig. 4:**
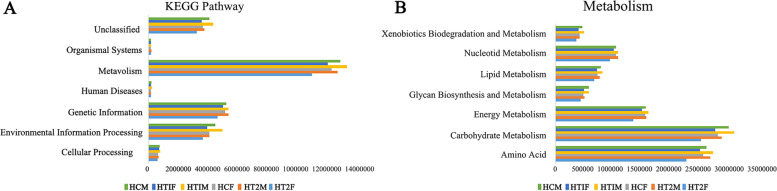
Analysis of gut microbial function in patients with Hashimoto’s thyroiditis. **A** KEGG analysis of gut microbes in patients with Hashimoto’s thyroiditis. **B** Analysis of metabolic function of gut microbes in patients with Hashimoto’s thyroiditis

Functional predictions of the gut microbiota were performed using PICRUSt, and the KEGG analysis results showed that metabolic function was the most abundant function in the gut microbiota. Metabolic function differed between males and females, and its relative abundance was lower in the HT2A group than in the HCA and HT1A groups in both males and females (Fig. 4A). Further analysis of the metabolism function revealed that carbohydrate metabolism, amino acid metabolism, and energy metabolism were the three most abundant functions, with carbohydrate metabolism and amino acid metabolism being stronger in males than in females and the carbohydrate metabolism function being weaker in the HT2A group than in the corresponding HCA and HT1A groups. The amino acid metabolism function was more consistent in males but was weaker in females in the HT2A group than in the corresponding HCA and HT1A groups (Fig. 4B).

### Association analysis of environmental factors affecting differences in gut microbes in patients with Hashimoto’s thyroiditis

**Fig. 5:**
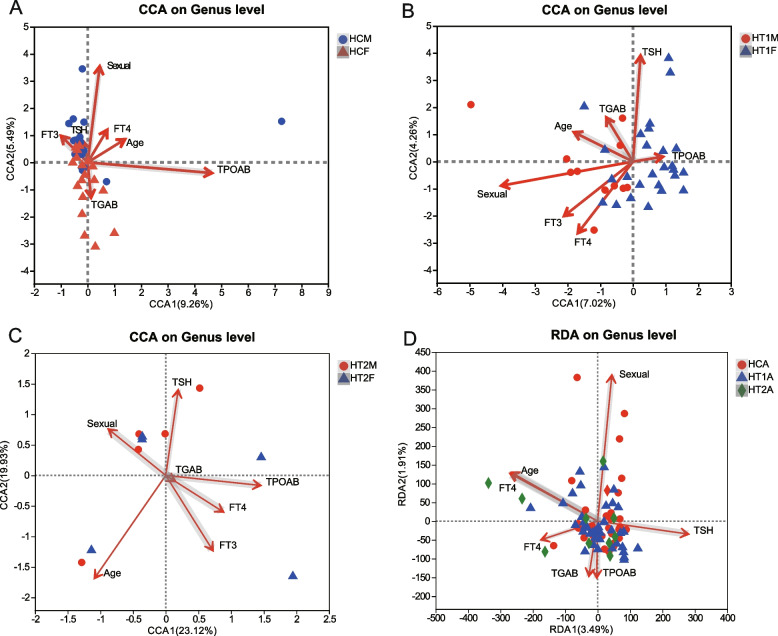
Association analysis of gut microbial environmental factors in patients with Hashimoto’s thyroiditis based on genus level. **A** CCA association analysis of gut microbes in healthy males and females. **B** CCA association analysis of gut microbes in males and females with Hashimoto’s thyroiditis and normal thyroid function. **C** CCA association analysis of gut microbes in males and females with Hashimoto’s thyroiditis and abnormal thyroid function. **D** RDA association analysis of gut microbes in healthy people, normal people with Hashimoto’s thyroiditis, and people with Hashimoto’s thyroiditis and abnormal thyroid function. HCA: healthy population, HT1A: Hashimoto’s thyroiditis with normal thyroid function, HT2A: Hashimoto’s thyroiditis with abnormal thyroid function, HCM: healthy male population, HCF: healthy female population, HT1M: Hashimoto’s thyroiditis in normal males, HT1F: Hashimoto’s thyroiditis in normal thyroid females, HT2M: Hashimoto’s thyroiditis in abnormal thyroid males, HT2F: Hashimoto’s thyroiditis in abnormal thyroid females. CCA: canonical correspondence analysis; RDA: redundancy analysis. Points with different colors or shapes in the figure represent groups of samples under different environments or conditions; dots are used in the figure to represent different samples, and arrows emanating from the origin represent different environmental factors. The length of the environmental factor arrows represents the degree of influence (explained amount) of the environmental factors on species data; the angle between the environmental factor arrows represents positive and negative correlations (acute angle: positive correlation; obtuse angle: negative correlation; right angle: no correlation with sex); the vertical distance from the sample point to the environmental factor arrow and the extended line represents the strength of the influence that an environmental factor has on the sample. The closer the distance between the sample point and the arrow, the stronger the effect of the environmental factor on the sample. In the analysis results, the size of the first axis of Axis_lengths is used to determine the choice of RDA or CCA. If it is greater than or equal to 3.5, the default CCA is displayed, and if it is less than 3.5, the default RDA is displayed

Using RDA/CCA to conduct environmental factor association analysis, the results showed that, in healthy HCA, the differences in gut microbes between males and females were mainly affected by gender and TPOAB, and gender was positively correlated with thyroid hormones FT3 and FT4 (Fig. 5A); however, in addition to gender, the hormones FT3, FT4 and TSH were found to play a role in the differences between males and females in the HT1A group, and gender still showed a positive correlation with hormones and age (Fig. 5B); furthermore, in the HT2A group, age, FT3, TPOAB, and TSH were found to play an important role. Gender was no longer the most important dominant factor and began to show a negative correlation with hormones FT3 and FT4 (Fig. 5C).Comprehensive analysis of the three groups found that gender was an important factor affecting the composition of gut microbes, but with the development of HT, hormones FT4, age, and TSH gradually begin to play an important regulatory role (Fig. 5D).

**Fig. 6:**
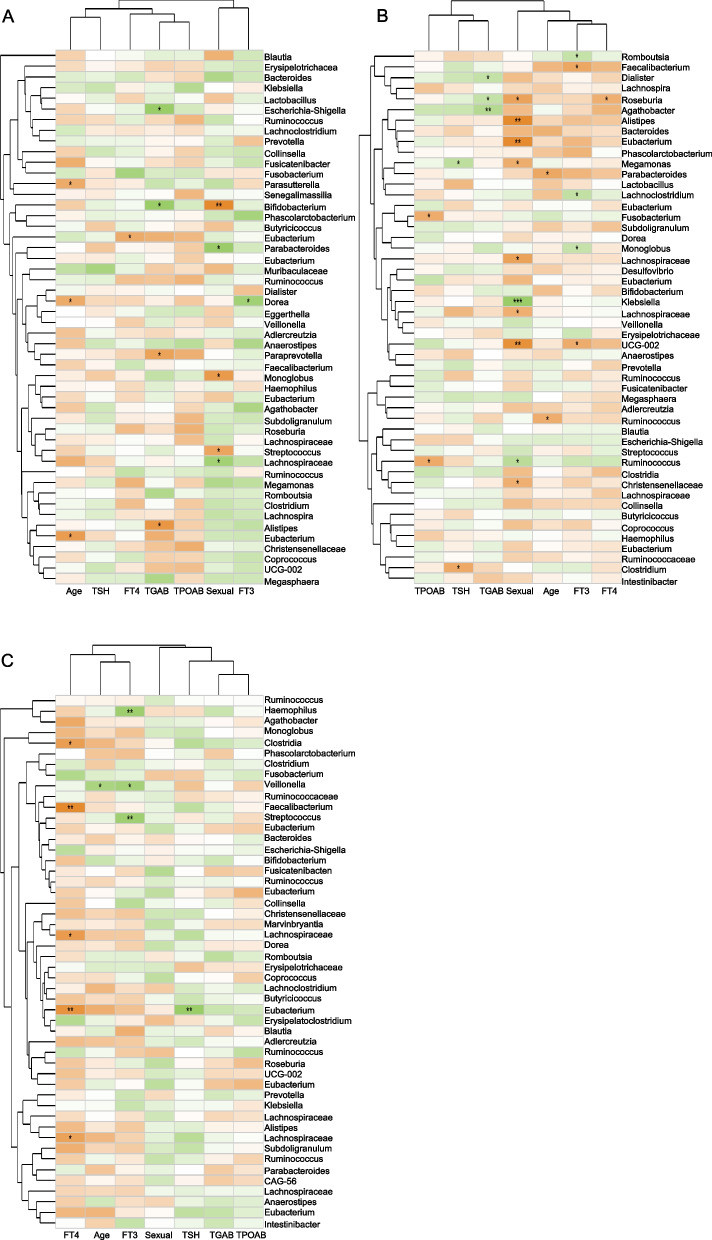
Heatmap analysis of gut microbes in Hashimoto’s thyroiditis patients based on Spearman correlation analysis. **A** Spearman correlation analysis heat map of gut microbes in healthy people. **B** Spearman correlation analysis heat map of gut microbes in Hashimoto’s thyroiditis and normal thyroid patients. **C** Heatmap of Spearman correlation analysis of gut microbes in patients with Hashimoto’s thyroiditis and abnormal thyroid function. FT4: free thyroxine, FT3: free triiodothyronine, Age: age, Sexual: gender, TSH: thyroid-stimulating hormone, TGAB: thyroglobulin antibody, TPOAB: thyroid peroxidase antibody. The above analysis is based on the analysis at the genus level. The X-axis and Y-axis in the figure are environmental factors and species, respectively, and the correlation R value and P value are obtained by calculation. The R value is displayed in different colors in the figure. If the *p* value is less than 0.05, it is marked with *. The legend on the right is the color interval of the different R values; the left and upper sides show the species and environmental factor clustering trees, respectively, * *p* ≤ 0.05, **p ≤ 0.01, ***p ≤ 0.001

The correlation heatmap and Spearman correlation analysis were further used to visualize the relationship between different species in the sample and environmental variables through correlation numerical visualization, and the correlation between microbial classification and environmental variables was evaluated. The results showed that in the HCA group, the most significantly related flora regulated by gender were *Bifidobacterium*, *Parabacteroide*, *Monoglobus*, *Streptococcus*, and unclassified_f__*Lachnospiraceae*, and very few were regulated by hormones (Fig. 6A); in the HT1A group, the most significant related flora were regulated by gender. For example, gender was positively correlated with *Klebsiella* and *Ruminococcus*_*gnavus*_group and negatively correlated with *Roseburia*, *Alistipes*, *Eubacterium*_*coprostanoligenes*_group, *Megamonas*, *Lachnospiraceae*, etc. However, in the HT1A group, the flora regulated by FT3, such as *Romboutsia*, *Faecalibacterium*, *Lachnoclostridium*, *Monoglobus*, etc., began to increase. Among them, FT3 was positively correlated with *Romboutsia*, *Lachnoclostridium,* and *Monoglobus,* but was negatively correlated with *Faecalibacterium*. In addition, TGAB also began to play an important role (Fig. 6B). In the HT2A group, no strains were significantly related to gender, but the genera regulated by FT3 and FT4, such as *Haemophilus*, *Veillonella*, and *Streptococcus,* which were significantly positively related to FT3,as well as norank_f__norank_o__*Clostridia*_UCG-014, *Faecalibacterium*, *Lachnospiraceae*_NK4A136_group, *Eubacterium*_*ruminantium*_group, and *Lachnospiraceae*_UCG-001, were negatively related to FT4 (Fig. 6C). These results further suggest that the hormones FT3 and FT4 play important roles in the regulation of gut microbes as HT develops.

## Discussion

In this study, we found that there were differences in the gut microorganisms between in healthy people HCA, HT1A with normal thyroid function in HT, and HT2A patients with hypothyroidism in HT, and these differences were enhanced as the severity of HT increased, indicating that HT and the consequent thyroid dysfunction have an important impact on the gut microbial composition. Studies have also found that gender has a strong impact on gut microbes. In healthy people, gender becomes the most important factor affecting gut microbial composition, and although hormonal effects gradually replace the role of gender with the development of HT, the impact of HT on gut microbes in different genders remains, suggesting that the impact caused by gender-based factors must be considered in the process of continuing in-depth study as well as in future gut microbiota-based interventions, so as to achieve more precise treatment and intervention.

### Differences in Gut microbiota between healthy people and patients with Hashimoto’s thyroiditis and their mediated functional correlations

This study found that *Lachnoclostridium*, *Holdemania*, *Eggerthella*, and*Bilophila* were enriched in the HCA group; *Akkermansia*, *Lachnoclostridium*, and *Holdemania* were enriched in HT1A; and *Akkermansia*, *Acetitomaculum*, *Shuttleworthia*, *Flavobacteriaceae*, unclassified_c__*Clostridia*, *Lachnospiraceae*_NC2004_group, and unclassified_o__*Oscillospirales* were enriched in HT1A. A previous study found that *Lachnoclostridium* was significantly enriched in rectal adenomas and could be used as a new biomarker for the non-invasive diagnosis of colorectal adenomas [37]. In addition, it was found to be significantly enriched in the guts of patients with cholestatic liver disease [38] and atherosclerosis [39]. The above results suggest that this bacterium maybe a biomarker that has a strong correlation with diseases, but it is interesting that this bacterium is enriched in HCA, and its abundance is higher than that in HT patients. However, the HT1A group still maintained a relatively high abundance, and the abundance of this bacteria was reduced in the HT2A group. While *Holdemania*, which was found to be enriched in HCA and HT1A patients, was found to be an important target of the Chinese herbal medicine known as Erchendecoction (ECD) and to enhance insulin sensitivity in previous studies [40], it was significantly decreased in the HT2A group. Additionally, it was found that *Bilophila*, a lipopolysaccharide-producing bacterium, exacerbates inflammation and leads to metabolic disturbances in HFD-mice on a high-fat diet in adulthood [41] and that improvements in body weight and glycolipid metabolism are inversely correlated with the relative abundance of *Bilophila*. These bacteria were also enriched in HCA, but decreased in HT2A. *Akkermansia* is a normal bacterium in the human gut and is a Mucin-degrading bacterium that is negatively correlated with obesity, diabetes, cardiovascular disease, and low-grade inflammation. It is a class of recently discovered star probiotics that has shown great potential for a number of applications [42] and has been found to be enriched in HT1A and HT2A. Likewise, *Lachnospiraceae*, which was enriched in HT1A and HT2A, were identified as being inversely associated with colitis and was able to protect mice from colitis-induced death [43]. *Shuttleworthia* was found to be enriched in systemic lupus erythematosus (SLE) gut and was significantly reduced after treatment [44]. The SLE is a typical autoimmune disease characterized by chronic inflammation and multiple organ damage, the patient’s self-tolerance is reduced, and autoantibodies continue to be produced. This disease and HT are both autoimmune diseases, thus the *Shuttleworthia* may possess the potential to be a biomarker of autoimmune diseases. In addition, gut microbes and their metabolites were found to play a key role in the development of hepatocellular carcinoma (HCC), and *Clostridia* was the dominant genus comprising the gut microbes in the HCC group, suggesting bacteria of this genus negatively regulate health [45].

Interestingly, some bacterial groups are strongly associated with disease, such as *Lachnoclostridium* and *Bilophila*, both of which were enriched in the HCA group, but decreased in the HT2A group; however, some probiotics, such as *Akkermansia* and *Lachnospiraceae*, were enriched in the HT2A group instead. Some disease-associated bacteria, such as *Shuttleworthia* and *Clostridia*, among others, were enriched in the HT2A group. The results suggest that although the gut microbiota of HT patients has changed, is the change in the direction of becoming healthier? Does it help the host to fight the disease, or does it promote the development of the disease? Conclusions cannot be drawn from the current data. In this study, genera were identified by high-throughput sequencing based on 16S rDNA. Previous studies have found that there may be great differences in the functions between different strains of the same strain [46], which is also the reason for the current situation. Therefore, the relationship between these strains and host health and disease needs further study.

### The effect of gender on the flora and its significance

*Bifidobacterium* is a bacterium that produces short-chain fatty acids (SCFAs), which degrade polysaccharides and dietary fiber. The relative abundance of *Bifidobacterium* is directly associated with improved body weight and glucose and lipid metabolism [41]. This group of bacteria has long been considered to be positively related to health. In this study, the abundance of *Bifidobacterium* in healthy people was lower than that in HT patients, and the abundance in females was much higher than it was in males. However, in HT patients, the reversal occurred in both HT1A and HT2A, and the abundance in males was higher than it was in females. The total abundance increased with as the HT levels increased. The levels of *Klebsiella* bacteria were higher in males than in females and showed an initial increasing trend and then a decreasing trend as the HT level became more aggravated. In the HT2A group in particular, HT2AF was significantly reduced. *Bifidobacterium* and *Klebsiella* are two types of bacteria that are very variable in healthy people and in HT patients, but it was interesting that the Spearman correlation analysis showed that these two types of bacteria were only regulated according to sex, especially *Bifidobacterium*, which was found both in patients and in healthy people of both sexes. A reversal in the species abundances determining the diversity was observed. At the same time, the results show that HT not only leads to differences in the gut microbiota between males and females, but also suggests that HT may have different effects and symptoms in males and females, indicating that gender factors need to be considered in the process of clinical intervention and treatment. Another interesting phenomenon is that *Bifidobacterium* and *Klebsiella* are two types of very typical microbes found in the gut microbiota. Among them, *Bifidobacterium* is usually related to intestinal health, while *Klebsiella* is an opportunistic pathogen that is often accompanied by disturbances in the gut microbiota, many of which are caused by external conditions. Previous studies have found that *Klebsiella* is involved in the regulation of the gut microbiota–immune system–brain axis, and the overgrowth of *Klebsiella* in the gastrointestinal tract may be directly related to the increase in the number of specific immune cells and the occurrence of neurological damage observed in premature infants [47]. In this study, it was found that the abundance of *Bifidobacterium* in the guts of HT patients increased with the development of the disease, while *Klebsiella* showed an initial increasing and then decreasing, especially in HT2F group was significantly lower than the healthy population. Is this the result of natural changes in the flora caused by hormone disorder? Or is it the active regulation taken by the body to protect itself and cope with the adverse effects of diseases, so as to use the immune system and other related metabolic systems? Or is it caused by the insufficient sample size of HT2A? The mechanism of this phenomenon deserves further study based on animal models.

### Regulation and significance of hormones on bacterial flora

This study found that hormones can significantly affect the gut microbiota of HT patients. In the HT1A group, FT3 was significantly positively correlated with *Romboutsia*, *Lachnoclostridium,* and *Monoglobus*, but was significantly negatively correlated with *Faecalibacterium*. In the HT2A group, FT3 was significantly positively correlated with *Haemophilus*, *Veillonella*, and Streptococcus, and FT4 was significantly negatively correlated with norank_f__norank_o__*Clostridia*_UCG-014, *Faecalibacterium*, *Lachnospiraceae*_NK4A136_group, *Eubacterium*_*ruminantium*_group, and *Lachnospiraceae*_UCG-001. Previous studies have found that *Romboutsia* can regulate the behavior of mice through neuroactive metabolites, indicating that it is related to autism spectrum disorder (ASD). A positive correlation was observed, and as FT3 decreased, the abundance of the bacteria also decreased, suggesting that thyroiditis-mediated gut microbes may affect autistic behavior in the host [48]. *Haemophilus* is negatively correlated with carotid atherosclerosis (CAS), and the abundance of this bacterium is decreased in the guts of CAS patients, suggesting that this bacterium may play an important role in regulating health [49]. In HT2A, FT3 was positively correlated with *Haemophilus*, suggesting that asFT3 decreases, the abundance of this bacteria is decreased, and the composition of the gut microbiota of patients begins to develop in an unhealthy direction. On the other hand, *Faecalibacterium* is one of the most important bacteria in the human gut microbiota as well as an important producer of butyric acid, playing important anti-inflammatory roles and protecting the digestive system from intestinal pathogens [50]. In the HT2A group, FT4 was negatively correlated with this bacterium. With the decreased of FT4, the abundance of *Faecalibacterium* increased, resulting in a protective effect on the intestinal tract. *Lachnospiraceae*, a class of probiotics that regulates colitis and helps the host to defend against disease [41], is negatively correlated with FT4, and with the decrease in FT4 in the HT2A group, the abundance of this bacteria also increased, resulting in a benefit to the host. Therefore, from the perspective of changes in gut microbiota regulated by hormones, some key probiotics are enriched in HT patients, while some bacteria associated with diseases are also enriched. The comprehensive result is that the function of gut microbiota changes in a beneficial or harmful direction, which still needs further study.

### Significance of this study

The results of this study indicate that both the occurrence and development of HT have an impact on the gut microbiota. Whether it is an overall change or regulation based on sex or hormones, two opposite results were produced: on the one hand, in the intestinal tracts of HT patients, the abundances of some beneficial bacteria in the tract increase, and on the other hand, some microorganisms associated with the disease also increase. What impact will this change pattern in the gut microbiota have on host physiology and health? Whether it further exacerbates the disease or helps to delay and fight the disease is currently unknown. Of course, we know that, as the microbiota is a micro-ecosystem, it is impossible to change simple probiotics or pathogenic bacteria so that the gut composition changes in a single direction. These changes are complex and are influenced by both probiotics or pathogenic bacteria. Therefore, in the future, when we study the function of gut microbes, we cannot solely consider the function caused by the changes in a certain type of strain, but also need to use systematic thinking to make comprehensive judgments. On the other hand, through this study, we found that gender is an important regulator of gut microbes and responds to HT. Therefore, in the future interventions and treatments of thyroiditis, in addition to paying attention to the role of hormones, gender-mediated functional heterogeneity must also be considered in its entirety. Another thing worth noting is that, although we have collected samples for more than three years, but as we set strict standards for the included samples, many HT patients with thyroid dysfunction who have been treated are excluded, which leads to difficulties in sample collection, so the number of samples in this group is too small. Although the number of cases in this group is small, we also found some significant phenomena that distinguish other groups. From the experimental data, the data of HT2A group conforms to the trend of gradual development of a disease from HCA to HT1A, and then to HT2A. This is a very interesting phenomenon, and it's also logical. These phenomena have a good guiding significance for the subsequent collection of large samples for in-depth research. Of course, in order to be more rigorous, we should continue to increase the collection of samples in the future to avoid false estimation of the true changes of gut microorganisms caused by insufficient sample size.

## Supplementary Information

**Additional file 1.** 

**Additional file 2.** 

**Additional file 3.** 

**Additional file 4.** 

**Additional file 5.**

## Data Availability

The datasets presented in this study can be found in online repositories. The names of the repository/repositories and accession number(s) can be found below: the NCBI Sequence Read Archive (SRA) database (http://www.ncbi.nlm.nih.gov/bioproject) and the accession number is PRJNA881637.
