# Supplementary material for: Analysis of gut microbiota diversity in Hashimoto’s thyroiditis patients
Source: BMC Microbiol. 2022 Dec 24;22:318. doi: 10.1186/s12866-022-02739-z (PMC9789560; doi:10.1186/s12866-022-02739-z)
Supplement: Supplementary file 1 — Additional file 1. [file 12866_2022_2739_MOESM1_ESM.docx]

**Supplementary Information**

| **Analysis of Gut Microbiota Diversity in Hashimoto’s Thyroiditis Patients** |
| --- |

**Jilai Liu^1^, Xuejun Qin^1^, Boxi Lin^1^, Jing Cui^1^, Juan Liao^1^, Fu Zhang^1,*^ and Qing Lin^1, *^**

^1^ Department of Clinical Laboratory, People's Hospital Affiliated of Fujian University of Traditional Chinese Medicine, Fuzhou, 350004, China; [450599054@qq.com (J.L.)](mailto:450599054@qq.com%20(J.L.)); [371038389@qq.com (X.Q.)](mailto:371038389@qq.com%20(X.Q.)); [linboxi0875@163.com (B.L.)](mailto:linboxi0875@163.com%20(B.L.)); [cuijing217@163.com (J.C.)](mailto:cuijing217@163.com%20(J.C.)); [liaojuan858@163.com](mailto:liaojuan858@163.com) (J.L.)

**^*^** Correspondence: [22581605@qq.com](mailto:22581605@qq.com) (F.Z.); [fjlinqing@126.com](mailto:fjlinqing@126.com) (Q.L.)

**This file includes:**

Supplementary Figures 1~17

Supplementary Table legends 1-4


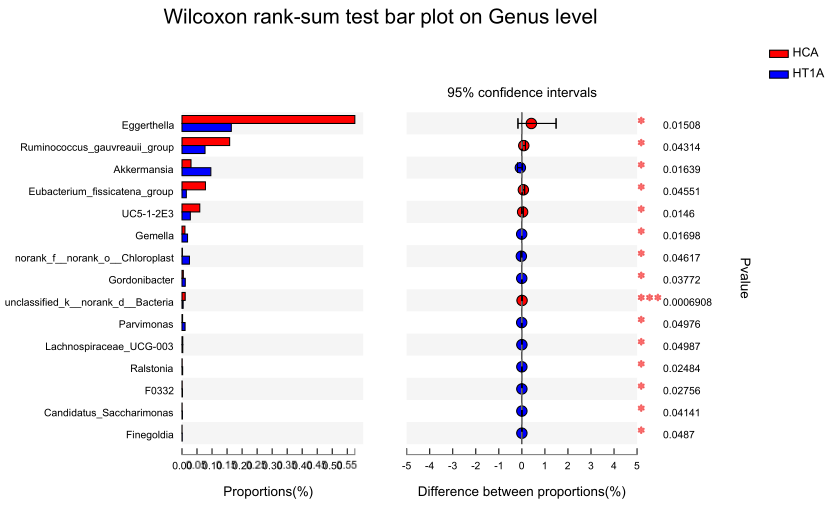


**FigureS1 Pairwise comparative analysis between HCA and HT1A.** HCA: healthy population, HT1A: Hashimoto’s thyroiditis with normal thyroid function. The pairwise comparative analysis was conducted by Wilcoxon method. The X axis represents different groups, the boxes with different colors represent different groups, and the Y axis represents the average relative abundance of a species in different groups. The *p* value indicated significant difference between samples, **p*≤0.05, ****p*≤0.001.


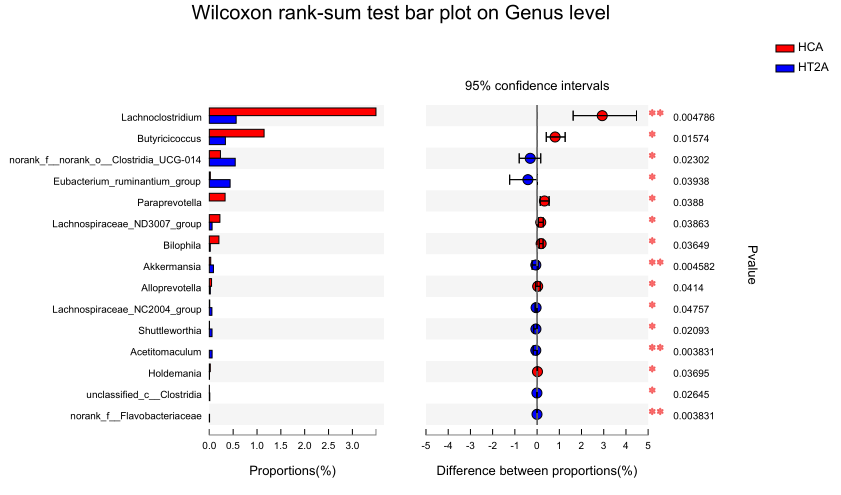


**Figure S2 Pairwise comparative analysis between HCA and HT2A.** HCA: healthy population, HT2A: Hashimoto’s thyroiditis with abnormal thyroid function. The pairwise comparative analysis was conducted by Wilcoxon method. The X axis represents different groups, the boxes with different colors represent different groups, and the Y axis represents the average relative abundance of a species in different groups. The *p* value indicated significant difference between samples, **p*≤0.05, ***p*≤0.01.


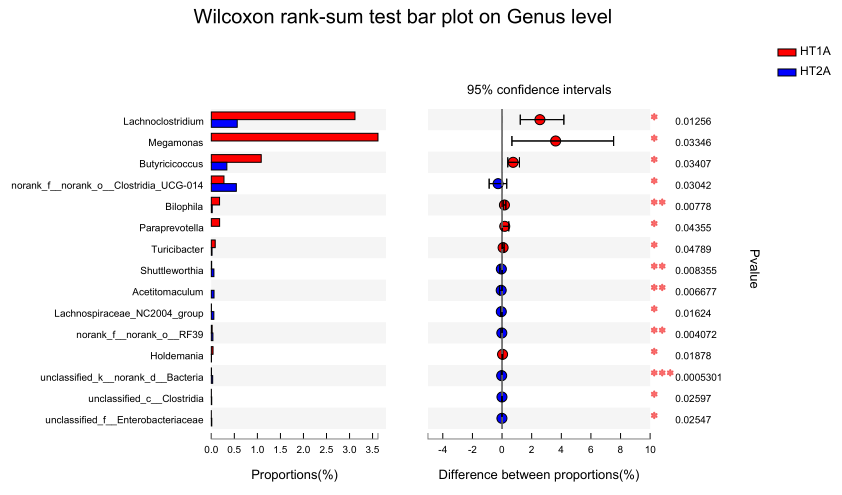


**Figure S3 Pairwise comparative analysis between HT1A and HT2A.** HT1A: Hashimoto’s thyroiditis with normal thyroid function, HT2A: Hashimoto’s thyroiditis with abnormal thyroid function. The pairwise comparative analysis was conducted by Wilcoxon method. The X axis represents different groups, the boxes with different colors represent different groups, and the Y axis represents the average relative abundance of a species in different groups. The *p* value indicated significant difference between samples, **p*≤0.05, ***p*≤0.01, ****p*≤0.001.


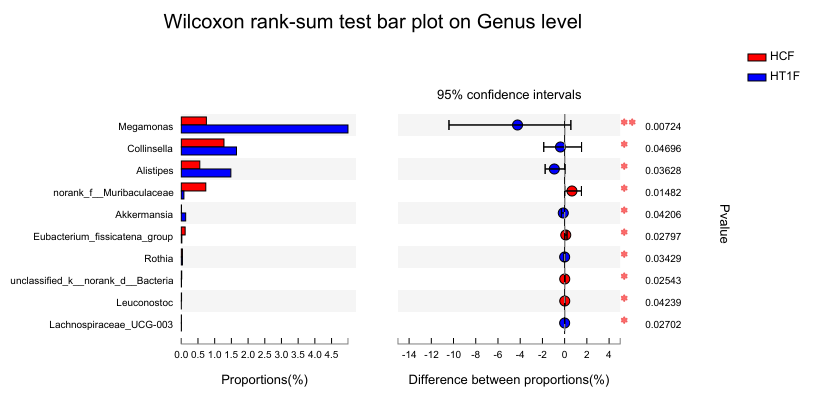


**Figure S4 Pairwise comparative analysis between HCF and HT1F.** HCF: healthy female population, HT1F: Hashimoto’s thyroiditis in normal thyroid females. The pairwise comparative analysis was conducted by Wilcoxon method. The X axis represents different groups, the boxes with different colors represent different groups, and the Y axis represents the average relative abundance of a species in different groups. The *p* value indicated significant difference between samples, **p*≤0.05, ***p*≤0.01.


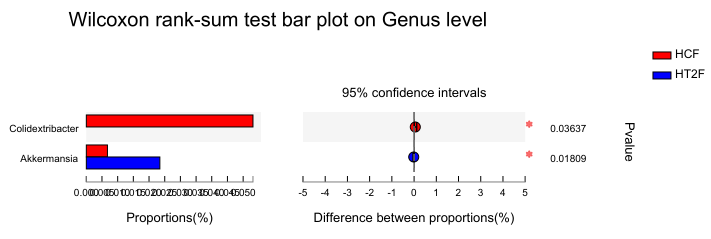


**Figure S5 Pairwise comparative analysis between HCF and HT2F.** HCF: healthy female population, HT2F: Hashimoto’s thyroiditis in abnormal thyroid females. The pairwise comparative analysis was conducted by Wilcoxon method. The X axis represents different groups, the boxes with different colors represent different groups, and the Y axis represents the average relative abundance of a species in different groups. The *p* value indicated significant difference between samples, **p*≤0.05.


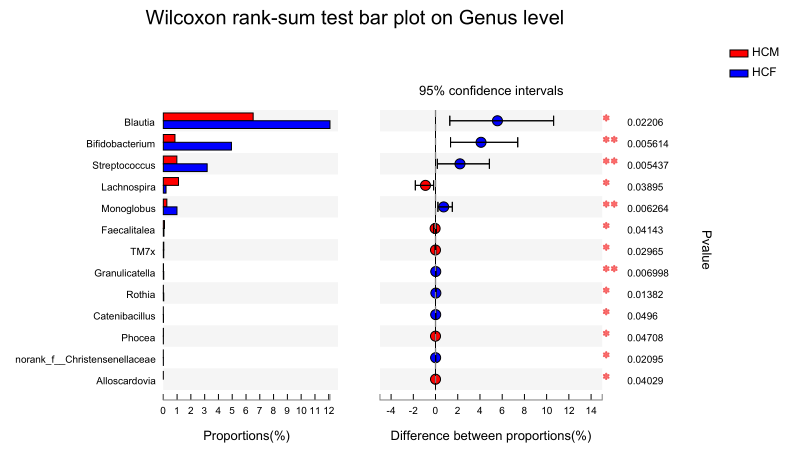


**Figure S6 Pairwise comparative analysis between HCM and HCF.** HCM: healthy male population, HCF: healthy female population. The pairwise comparative analysis was conducted by Wilcoxon method. The X axis represents different groups, the boxes with different colors represent different groups, and the Y axis represents the average relative abundance of a species in different groups. The *p* value indicated significant difference between samples, **p*≤0.05, ***p*≤0.01.


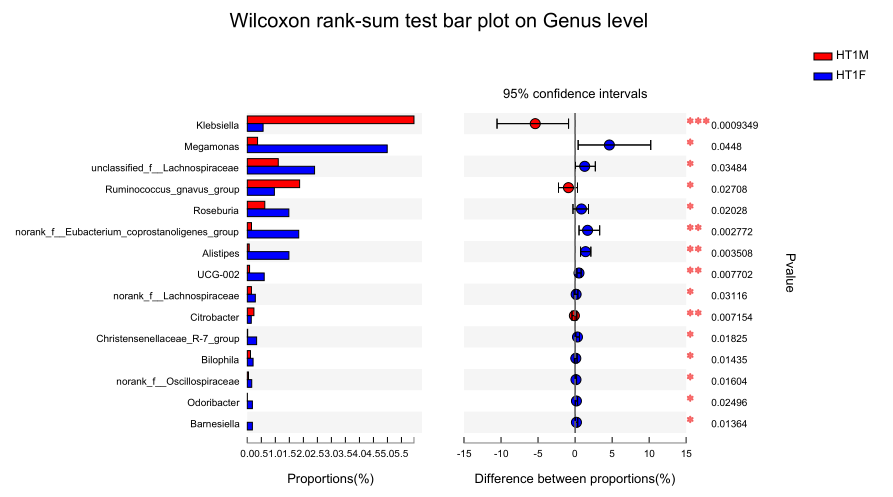


**Figure S7 Pairwise comparative analysis between HT1M and HT1F.** HT1M: Hashimoto’s thyroiditis in normal males, HT1F: Hashimoto’s thyroiditis in normal thyroid females. The pairwise comparative analysis was conducted by Wilcoxon method. The X axis represents different groups, the boxes with different colors represent different groups, and the Y axis represents the average relative abundance of a species in different groups. The *p* value indicated significant difference between samples, **p*≤0.05, ***p*≤0.01, ****p*≤0.001.


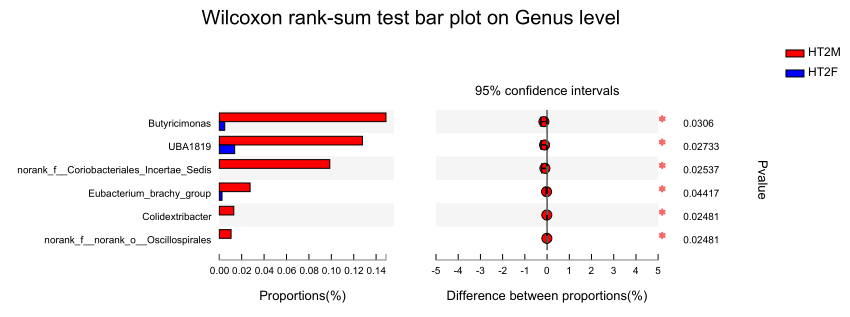


**Figure S8 Pairwise comparative analysis between HT2M and HT2F.** HT2M: Hashimoto’s thyroiditis in abnormal thyroid males, HT2F: Hashimoto’s thyroiditis in abnormal thyroid females. The pairwise comparative analysis was conducted by Wilcoxon method. The X axis represents different groups, the boxes with different colors represent different groups, and the Y axis represents the average relative abundance of a species in different groups. The *p* value indicated significant difference between samples, **p*≤0.05.


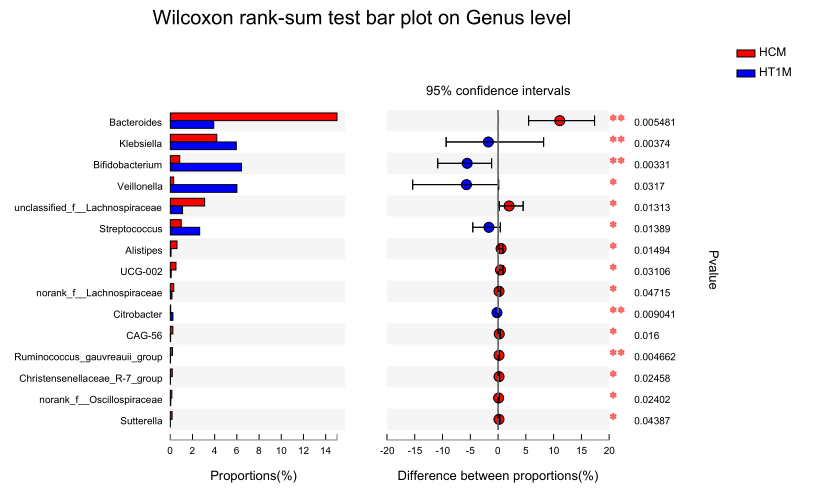


**Figure S9 Pairwise comparative analysis between HCM and HT1M.** HCM: healthy male population, HT1M: Hashimoto’s thyroiditis in normal males. The pairwise comparative analysis was conducted by Wilcoxon method. The X axis represents different groups, the boxes with different colors represent different groups, and the Y axis represents the average relative abundance of a species in different groups. The *p* value indicated significant difference between samples, **p*≤0.05, ***p*≤0.01.


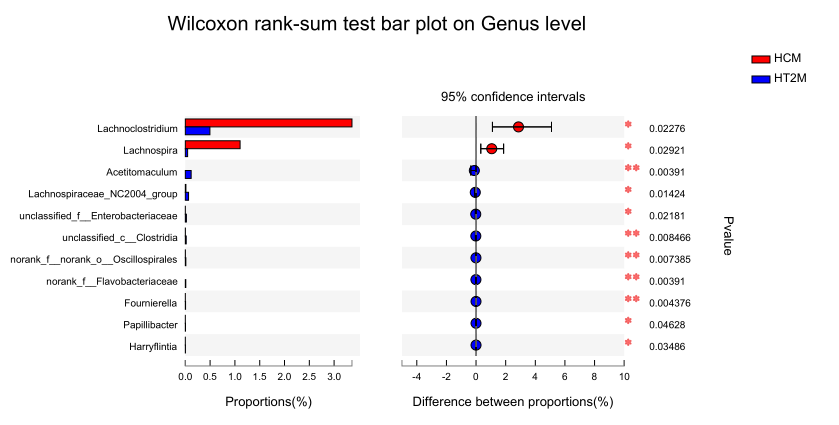


**Figure S10 Pairwise comparative analysis between HCM and HT2M.** HCM: healthy male population, HT2M: Hashimoto’s thyroiditis in abnormal thyroid males. The pairwise comparative analysis was conducted by Wilcoxon method. The X axis represents different groups, the boxes with different colors represent different groups, and the Y axis represents the average relative abundance of a species in different groups. The *p* value indicated significant difference between samples, **p*≤0.05, ***p*≤0.01.


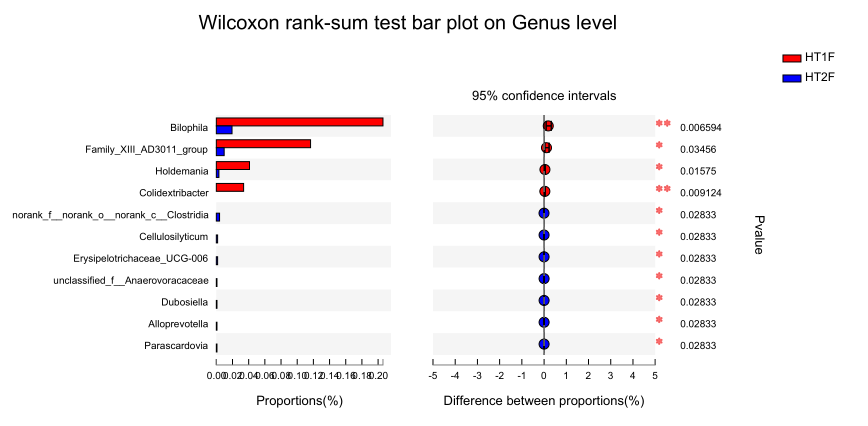


**Figure S11 Pairwise comparative analysis between HT1F and HT2F.** HT1F: Hashimoto’s thyroiditis in normal thyroid females, HT2F: Hashimoto’s thyroiditis in abnormal thyroid females. The pairwise comparative analysis was conducted by Wilcoxon method. The X axis represents different groups, the boxes with different colors represent different groups, and the Y axis represents the average relative abundance of a species in different groups. The *p* value indicated significant difference between samples, **p*≤0.05, ***p*≤0.01.


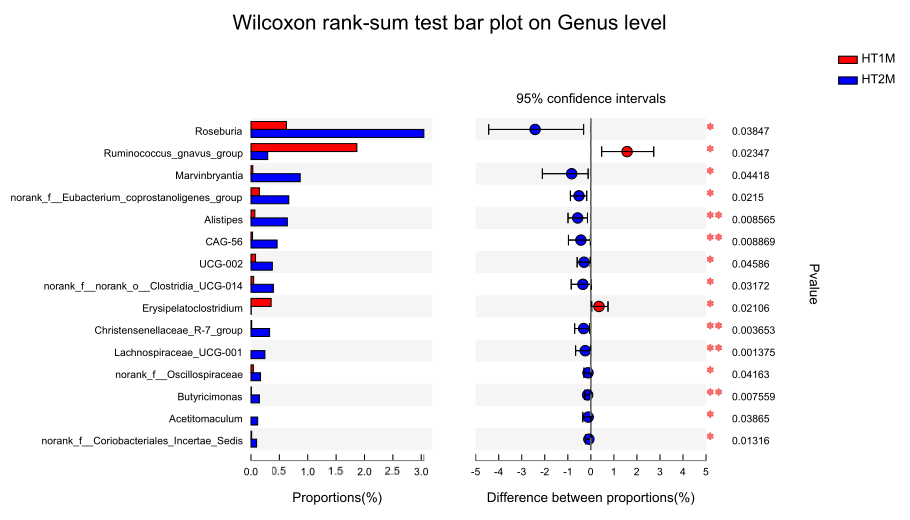


**Figure S12 Pairwise comparative analysis between HT1M and HT2M.** HT1M: Hashimoto’s thyroiditis in normal males, HT2M: Hashimoto’s thyroiditis in abnormal thyroid males. The pairwise comparative analysis was conducted by Wilcoxon method. The X axis represents different groups, the boxes with different colors represent different groups, and the Y axis represents the average relative abundance of a species in different groups. The *p* value indicated significant difference between samples, **p*≤0.05, ***p*≤0.01.


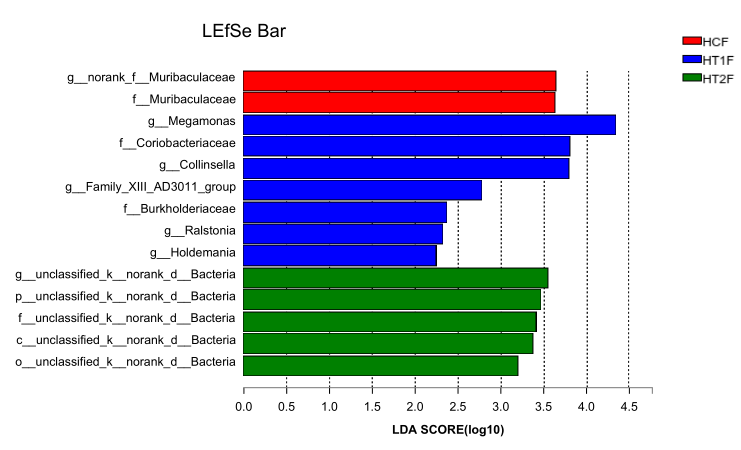


**Figure S13LEFSe analysis among HCF, HT1F and HT2F.** HCF: healthy female population, HT1F: Hashimoto’s thyroiditis in normal thyroid females, HT2F: Hashimoto’s thyroiditis in abnormal thyroid females. The linear regression analysis (LDA) discrimination column chart is used to count the microbial groups that have significant effects in multiple groups. The LDA score obtained through LDA analysis, the greater the LDA score, the greater the impact of species abundance on the difference effect.


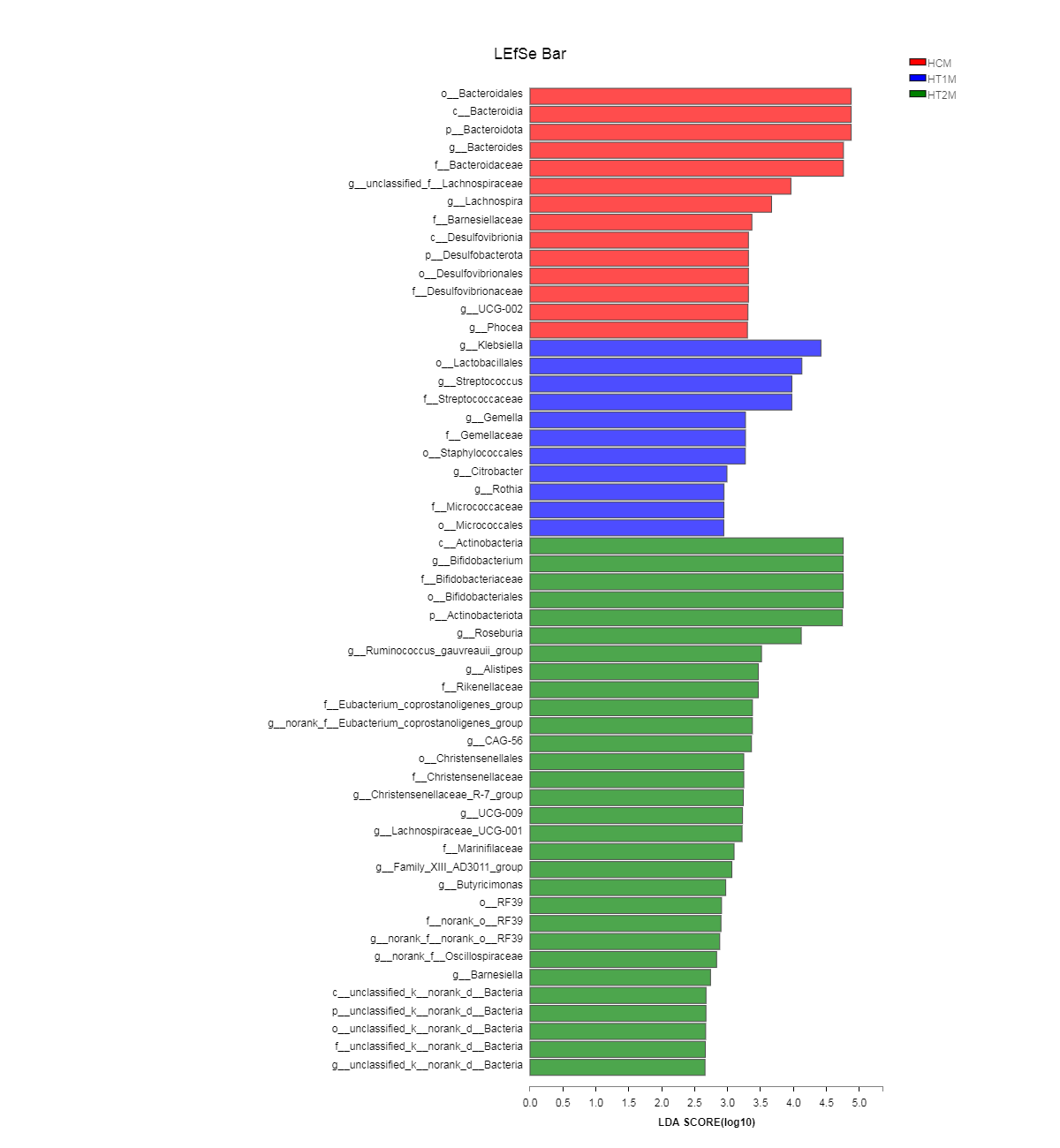


**Figure S14 LEFSe analysis among HCF, HT1M and HT2M.** HCF: healthy female population, HT1M: Hashimoto’s thyroiditis in normal males, HT2M: Hashimoto’s thyroiditis in abnormal thyroid males. The linear regression analysis (LDA) discrimination column chart is used to count the microbial groups that have significant effects in multiple groups. The LDA score obtained through LDA analysis, the greater the LDA score, the greater the impact of species abundance on the difference effect.


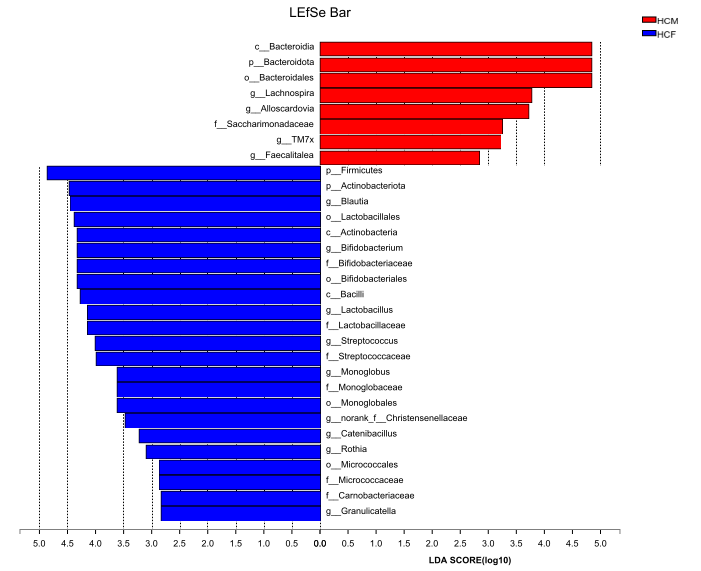


**Figure S15 LEFSe analysis between HCM and HCF.** HCM: healthy male population, HCF: healthy female population. The linear regression analysis (LDA) discrimination column chart is used to count the microbial groups that have significant effects in multiple groups. The LDA score obtained through LDA analysis, the greater the LDA score, the greater the impact of species abundance on the difference effect.


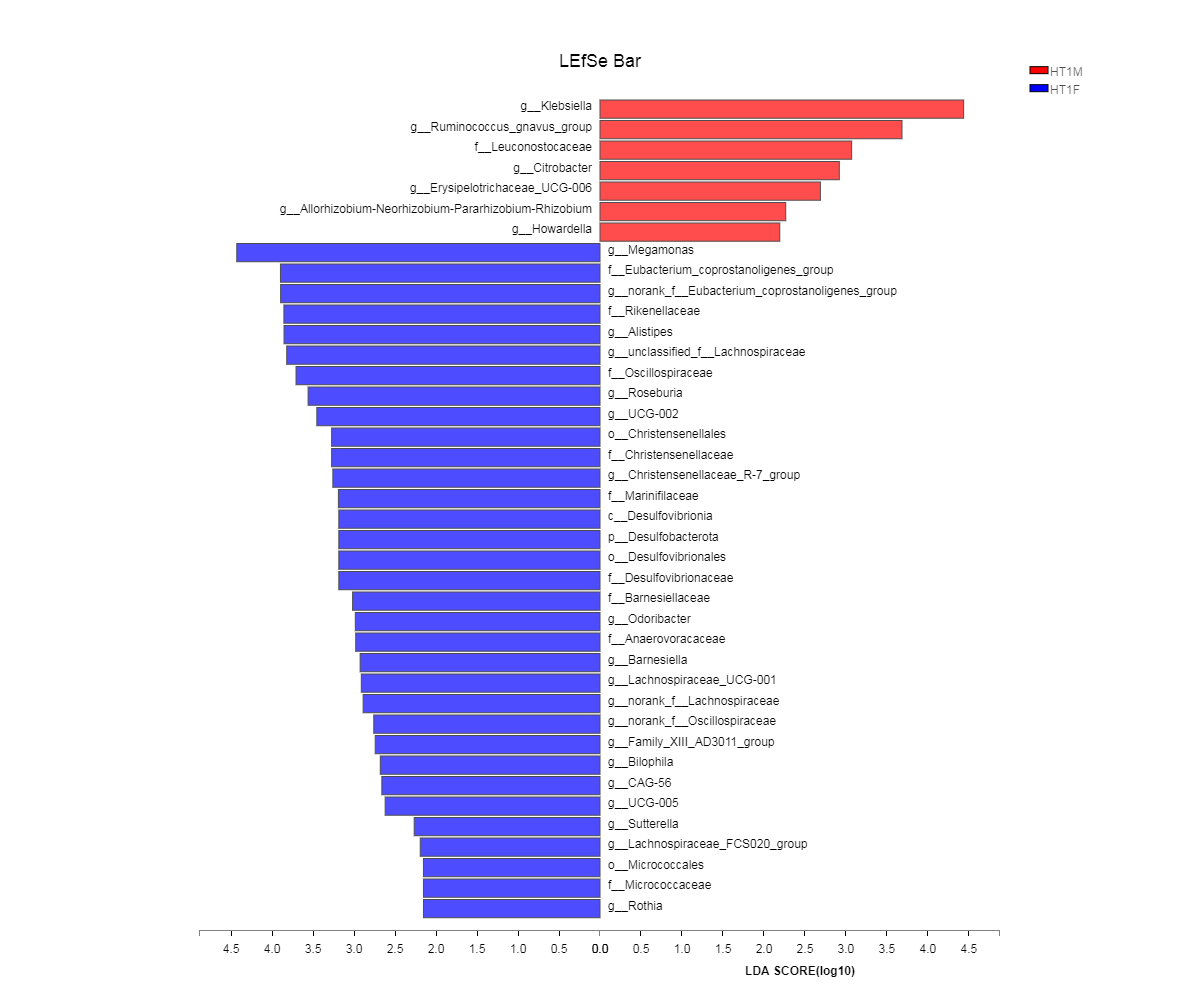


**Figure S16 LEFSe analysis between HT1M and HT1F.** HT1M: Hashimoto’s thyroiditis in normal males, HT1F: Hashimoto’s thyroiditis in normal thyroid females. The linear regression analysis (LDA) discrimination column chart is used to count the microbial groups that have significant effects in multiple groups. The LDA score obtained through LDA analysis, the greater the LDA score, the greater the impact of species abundance on the difference effect.


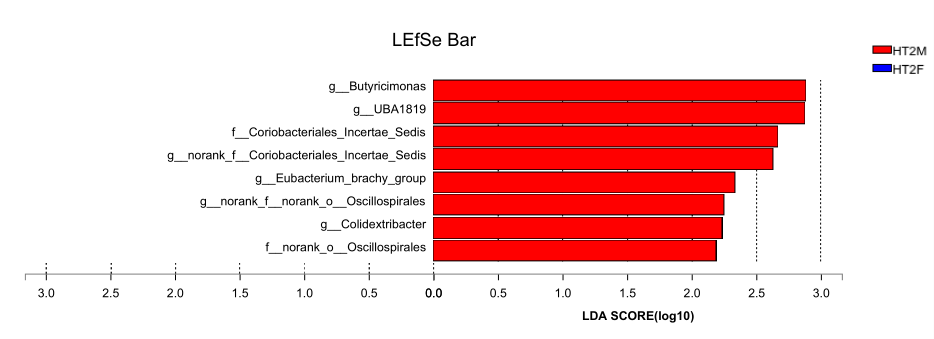


**Figure S17 LEFSe analysis between HT2M and HT2F.** HT2M: Hashimoto’s thyroiditis in abnormal thyroid males, HT2F: Hashimoto’s thyroiditis in abnormal thyroid females. The linear regression analysis (LDA) discrimination column chart is used to count the microbial groups that have significant effects in multiple groups. The LDA score obtained through LDA analysis, the greater the LDA score, the greater the impact of species abundance on the difference effect.

**Supplementary Table legends:**

**Table S1: The high-throughput sequencing information under MiSeq PE300 in Illumina of the 89 samples.**

**Table S2: OTUs and the species annotation information of the 89 samples.**

**Table S3: Alpha diversity of Chao 1, Shannon, Simpson, Sobs, ACE calculated by Mothur.** HCA: healthy population, HT1A: Hashimoto’s thyroiditis with normal thyroid function, HT2A: Hashimoto’s thyroiditis with abnormal thyroid function.

**Table S4: PICRUSt based KEGG analysis of the gut microbiota in different groups.** HCM: healthy male population, HCF: healthy female population, HT1M: Hashimoto’s thyroiditis in normal males, HT1F: Hashimoto’s thyroiditis in normal thyroid females, HT2M: Hashimoto’s thyroiditis in abnormal thyroid males, HT2F: Hashimoto’s thyroiditis in abnormal thyroid females.
